# Abnormal Brain Glucose Metabolism in Papillary Thyroid Cancer Patients 4 Weeks After Withdrawal of Levothyroxine: A Cross-Sectional Study Using ^18^F-FDG PET/CT

**DOI:** 10.3389/fendo.2021.595933

**Published:** 2021-03-11

**Authors:** Shu-qi Wu, Fang Feng, Ren-jian Zou, Hong-liang Fu, Jia-wei Sun, Xi-ze Jia, Ya-fu Yin, Hui Wang

**Affiliations:** ^1^ Department of Nuclear Medicine, Xinhua Hospital, Shanghai Jiaotong University School of Medicine, Shanghai, China; ^2^ School of Information and Electronics Technology, Jiamusi University, Jiamusi, China; ^3^ Institute of Psychological Sciences, Hangzhou Normal University, Hangzhou, China; ^4^ Zhejiang Key Laboratory for Research in Assessment of Cognitive Impairments, Hangzhou, China

**Keywords:** papillary thyroid carcinoma, regional cerebral glucose metabolism, hypothyroidism, mood disorders, PET/CT

## Abstract

**Background:**

There is no doubt that thyroid dysfunction is associated with psychiatric disorders. A large amount of thyroid carcinoma patients displayed mood disorders after the withdrawal of levothyroxine (LT4). However, it is unclear whether the disorders are related to the transient withdrawal of LT4, and if yes, what the possible underlying mechanism is. This study aims to investigate the abnormal regional cerebral glucose metabolism (rCMRglu) in a group of papillary thyroid cancer (PTC) patients without LT4 for 4 weeks and prove the relationship between the abnormal rCMRglu with depression and anxiety.

**Methods:**

Brain ^18^F-FDG PET/CT data of 38 consecutive PTC patients with high/intermediate-risk from June 2016 to December 2017 have been analyzed. Of the 38 patients, 23 are in the LT4 withdrawal group (WG) and 15 in the LT4 replacement group (RG). These patients were also evaluated for depressive and anxiety symptoms within 24 h after the scans based on the Hamilton Depression Rating Scale (17 items, HRDS-17) and the Hamilton Anxiety Rating Scale (HAMA) respectively.

**Results:**

Thirty-eight patients (12 men, 26 women; age range, 25–69 years; mean age, 45.8 years) were selected in the study. Compared with the RG, patients in WG showed depression and anxiety with higher total scores of HRDS-17 and HAMA (14.7 ± 5.8 *vs* 3.8 ± 5.5, t = −5.74, p = 0.00; 9.3 ± 4.3 *vs* 2.7 ± 4.1, t = −4.74, p = 0.00, respectively). In the brain glucose metabolism analysis, the WG patients showed lower rCMRglu in Occipital_Mid_R and Postcentral_L. On the other hand, data illustrated significant rCMRglu increases in the Frontal_Sup_Orb_L. Compared with the healthy group (HG), the rCMRglu of the Postcentral_L and Precuneus_L showed hypoactivity, but the Hippocampus_R and the Temporal_Inf_L showed hyperactivity. This analysis yielded a significant correlation between abnormal rCMRglu with the free thyroxine level, the serum thyroid-stimulating hormone level, HRDS-17, and HAMA scores.

**Conclusions:**

The findings showed that more PTC patients exhibited depression and anxiety after LT4 withdrawal for 4 weeks. More attention should be paid to these hypothyroid patients while they were in the hospital. Such a short-term LT4 withdrawal also likely induced abnormal rCMRglu. Our study attempts to explain the possible mechanism of mood disorders related to transient hypothyroidism.

## Introduction

The association between thyroid function and psychiatric disorders was described more than 200 years ago (1). Overt hypothyroidism is a major cause of mood disorder (2). Even subclinical hypothyroidism was connected with depression, especially in young patients (3). Early studies observed abnormal regional cerebral glucose metabolism (rCMRglu) or cerebral blood flow (CBF) in patients under hypothyroidism (4–7). However, only a few studies focused on rCMRglu in thyroid carcinoma (TC) patients under transient hypothyroidism (8).

With the rapid increase of thyroid cancer and the unavailability of recombinant human thyrotropin (rhTSH) and triiodothyronine (T3) in mainland China, the experts of the Chinese Society of Nuclear Medicine (CSNM) typically recommends a short-time withdrawal from thyroid hormone (levothyroxine, Euthyrox, Levothyroxine Sodium, Merck Sdn Bhd, Germany) for post-treatment assessment or preparing for the iodine 131 (^131^I) ablation of TC patients in clinical practices (9). Consequently, we observed a significant amount of patients began to suffer mood disorders. With advances in brain imaging technology, a combination of endocrine and psychological testing as well as PET scan can provide insight into the underlying mechanisms of the functional neuroendocrine relationships (10). Therefore, the authors attempt to apply similar technics to study the relationship between hypothyroid-related mood disorder and abnormal rCMRglu in transient hypothyroidism papillary thyroid carcinoma (PTC) patients.

To the authors’ knowledge, this is the first clinical PET/CT study comparing the glucose metabolism of the brain in age, gender, education, body mass index (BMI), pTNM stage, and risk category-matched PTC patients with or without thyroid hormones for 4 weeks in mainland China. We performed this cross-sectional study to investigate whether 4 weeks of transient hypothyroidism by LT4 withdrawal could cause mood disorders and changes in rCMRglu on PET/CT.

## Materials and Methods

### Subjects

**Figure 1:**
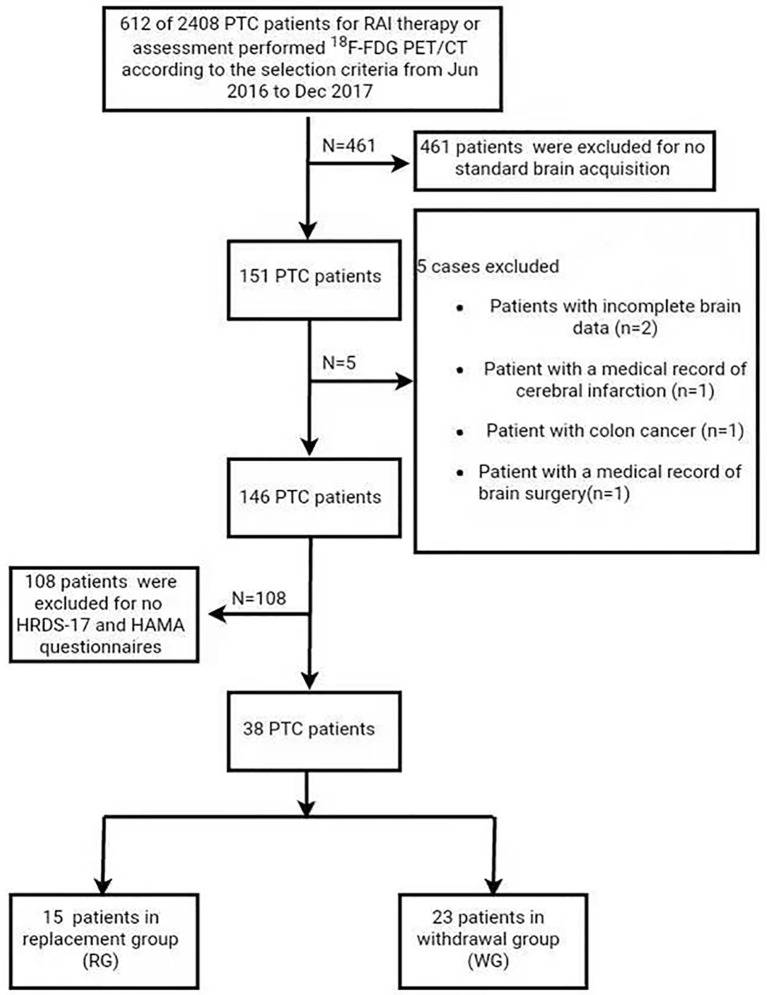
Flow chart of patients included in the current study. PTC, papillary thyroid carcinoma.

This retrospective study was approved by the Ethics Committee of Xin Hua Hospital Affiliated to Shanghai Jiaotong University School of Medicine (Approval No. XHEC-D-2019-117). Informed consent was obtained for all the patients/healthy participants for the publication of any potentially identifiable images, or data included in this article. From June 2016 to December 2017, 38 pathology-proofed PTC patients (12 men, 26 women; age range, 25–69 years; mean age, 45.8 years) who were standardly performed brain ^18^F-FDG PET/CT were analyzed in the study (**Figure 1**). Among them, 23 were in the LT4 withdrawal group (WG) and 15 in the LT4 replacement group (RG).

The inclusion criteria are: 1) all patients received total thyroidectomy, and the histological results were PTC; 2) Standard brain 18F-FDG PET/CT was performed in selected patients with high/intermediate-risk according to the guidelines of the American Thyroid Association (11) under standardized conditions described in the study; 3) HRDS-17 and HAMA questionnaires were evaluated within 24 h and thyroid function testing was performed within 3 days after the scans; 4) no known history of other endocrine problems and other malignant tumor diseases; 5) brain CT or MRI was performed for all patients to affirm no previous trauma or surgery of the brain.

The exclusion criteria are: 1) any history of alcohol or drug dependence; 2) medical records with diagnoses of psychiatric disorders and/or related medications; 3) severe medical conditions, such as organic brain disorders and neurological diseases including cerebrovascular diseases and seizures; 4) histories of head trauma; 5) serious somatic illnesses; 6) current pregnancy or breastfeeding.

We enrolled another 34 healthy subjects (7 men, 27 women; age range, 27–82 years; mean age, 48.97 years) from the same period as a healthy group (HG), following the 1:1 pairing rule for age and sex factors. The HG subjects were in euthyroid status during the sampling period and have given their written consents for participation in this study.

All of the selected PTC and healthy subjects were born in mainland China and of Chinese Han ethnicity. All were right-handed.

### Clinical Assessment

We recorded clinical information for each subject, including gender, body weight, height, years of education and career, pTNM stage, risk category, and thyroglobulin and thyroglobulin antibody levels. BMI was calculated. Serum levels of triiodothyronine (T3), free triiodothyronine (FT3), thyroxine (T4), free thyroxine (FT4), and TSH were also measured in blood samples using a time-resolved immunofluorometric assay (Any test, Sym-Bio Life Science Co., Ltd., Shanghai, China). Both HRDS-17 and HAMA tables, which were validated for use in mainland China (12, 13), were printed in Chinese and evaluated by a trained doctor who was blinded to the patients’ information. All subjects completed the scale assessments satisfactorily. The total HRDS-17 score has a range from 0 to 68, with a score higher than 17 reflecting moderate/severe depressed status (12–14). The HAMA value is used for assessing anxiety symptoms. The total score has a range from 0 to 64, with a score higher than 7 indicating increased anxiety (13).

### Brain ^18^F-FDG PET/CT Image Acquisition

^18^F-FDG PET/CT was performed on all subjects using a PET/CT scanner (Biograph mCT64, Siemens Medical Systems, Knoxville, TN, USA) in the 3D mode 45 min after intravenous injection of FDG (5.55 MBq/kg). Each patient was asked to fast for at least 6 h to ensure a serum glucose concentration of less than 150 mg/dl. After the injection, the subjects rested lying in a quiet, dimly lit environment. He or she was then evaluated with arms down and eyes closed in a dark and quiet examination room. The PET scan lasted for 10 min for each subject. A CT transmission scan was first obtained for attenuation correction (X-ray tube tension of 120 kV, current of 100–250 mAs, rotation time 0.8 s, and slice thickness 3 mm). Hanning filters and TureX (HD-PET) were used during image reconstruction. We used normalized count images to measure relative changes in regional glucose metabolism.

### Data Processing

Preprocessing of PET data was performed using the statistical parametric mapping 5 (SPM5, the Wellcome Department of Neurology, London U.K.) in MATLAB R2006a (MathWorks Inc., Sherborn, MA, USA). The PET images were spatially normalized to the SPM PET template (Montreal Neurological Institute, McGill University, Montreal, Canada), resliced with a voxel size of 2 × 2 × 2 mm^3^, and smoothed with a 10 mm full-width half-maximum isotropic Gaussian kernel. For standardization purposes, each voxel in the PET image was then divided by the global mean value of each individual (15).

### Statistical Analysis

To compare the PET maps between WG with RG and WG with HG respectively, two-sample t-tests were performed to identify the regions with significant differences with adjustments for age and sex. The statistical threshold was set at a threshold of individual voxel p < 0.005 at the voxel level and clusters size >150 voxels. The Pearson correlation analysis was performed with clinical/physiological/biochemical characteristics of the patients (including TSH, FT3, FT4 levels, the score of the HRDS-17 and HAMA) while adjusting for age and sex. The correlations were considered significant at a threshold of individual voxel p < 0.005, clusters size >150 voxels. The analyses were performed using RESTplus (16).

Differences in demographic and clinical variables between different groups were examined with independent t-test and chi-square test for sex. Fisher`s exact test for stage and risk category. A p value <0.05 (two-tailed) was considered statistically significant. All statistical analyses were conducted with SPSS software, version 22.0 (SPSS Inc., Chicago, IL, USA).

## Results

### Clinical Features

Thirty-eight consecutive patients with papillary thyroid carcinoma confirmed by pathology after total thyroidectomy were recruited in the study. Tumor staging was defined according to AJCC TNM, seventh edition (11); 18 patients were in stage I, 12 patients were in stage III, and 8 patients were in stage IV. Initial American Thyroid Association (ATA) risk stratification indicated intermediate-risk in 27/38 of the patients (71.1%) and high-risk in 11/38 of the patients (28.9%) based on clinical risk stratification for thyroid cancer. The demographic and clinical characteristics of the participants were presented in **Table 1**. There was no significant difference in age (p = 0.31), sex ratio (p = 0.37), years of education (p = 0.86), and BMI (p = 0.15) between the two groups. The pTNM stage and risk category were also compared using Fisher`s exact test (p = 0.35 and 0.07, respectively). There was no significant difference in Tg and TgAb (p = 0.25 and 0.09, respectively). As all the patients in the WG had clinically diagnosed hypothyroidism, the serum levels of T3 (t = −10.36, p = 0.00), T4 (t = −18.25, p = 0.00), FT3 (t = −5.66, p = 0.00), and FT4 (t = −17.62, p = 0.00) were lower than those in the RG, whereas the TSH level (t = 11.98, p = 0.00) was higher.

**Table 1:** Demographic and clinical characteristics of the study participants.

	RG (n = 15)	WG (n = 23)	Test^a^
**Age, year**	48.3 ± 13.3	44.1 ± 11.3	t = −1.03, p = 0.31
**Gender (male/female)**	6/9	6/17	χ^2^ = 0.81, p = 0.37
**Education, year**	11.3 ± 3.9	11.0 ± 4.7	t = −0.18, p = 0.86
**BMI**	25.8 ± 3.7	24.0 ± 3.6	t = −1.47, p = 0.15
**HRDS-17**	3.8 ± 5.5	14.7 ± 5.8	t = −5.74, p = 0.00
**HAMA**	2.7 ± 4.1	9.3 ± 4.3	t = −4.74, p = 0.00
**pTNM stage (I/III/IV)**	6/4/5	12/8/3	p = 0.35^b^
**Risk category (high/intermediate-risk)**	7/8	4/19	p = 0.07^b^
**T3, nmol/L (range**^c^)	1.1 ± 0.2 (1.0–1.2)	0.3 ± 0.3 (0.2–0.4)	t = −10.36, p = 0.00
**T4, ng/ml (range)**	109.2 ± 14.0 (84.7–140.3)	14.5 ± 16.60 (0.01–71.4)	t = −18.25, p = 0.00
**FT3, pmol/L (range)**	4.1 ± 0.9 (3.3–7.0)	2.2 ± 1.1 (0.1–4.0)	t = −5.66, p = 0.00
**FT4, pmol/L (range)**	18.1 ± 3.4 (14.2–25.7)	1.8 ± 2.3 (0.01–9.0)	t = −17.62, p = 0.00
**TSH, uIU/ml (range)**	0.5 ± 0.6 (0.01–2.04)	123.0 ± 49.0 (39.68–172.5)	t = 11.98, p = 0.00
**Tg, ng/ml (range)**	7.1 ± 8.5 (0.1–28.9)	16.3 ± 19.5 (0.1–83.8)	t = 1.17, p = 0.25
**TgAb, IU/ml (range)**	10.8 ± 4.7 (1.0–18.2)	8.3 ± 8.2 (1.3–42.0)	t = 1.72, p = 0.09

a Independent t-test for continuous variables and chi-square test for sex.

b Fisher’s exact test.

c range: from minimum to maximum.

RG, levothyroxine replacement group; WG, levothyroxine withdrawal group; BMI, body mass index; HRDS-17, 17 items of Hamilton Depression Rating Scale; HAMA, Hamilton Anxiety Rating Scale; T3, triiodothyronine; T4, thyroxine; TSH, thyroid-stimulating hormone; Tg, thyroglobulin; TgAb, thyroglobulin antibody.

**Table 2** shows the primary clinical information of the WG and HG. The mean age in the HG was higher than that in the WG, but there was no statistically significant difference (49.0 *vs.* 44.1, t = 1.4, p = 0.17). Moreover, the sex ratio in the two groups was not significantly different.

**Table 2:** The clinical information of HG and WG.

	HG (n = 34)	WG (n = 23)	Test
**Age, year**	49.0 ± 13.7	44.1 ± 11.3	t = 1.40, p = 0.17
**Gender (Male/female)**	7/27	6/17	χ^2^ = 0.24, p = 0.63
**Education, year**	11.0 ± 3.3	11.0 ± 4.7	t = 0.00, p = 1.00
**BMI**	22.9 ± 2.6	24.0 ± 3.6	t = −1.29, p = 0.20

Independent t-test for continuous variables and chi-square test for gender.

### HRDS-17 and HAMA Values in the WG and RG

The total scores of HRDS-17 were 14.7 ± 5.8 in the WG and 3.8 ± 5.5 in the RG, with a significant difference between the two groups (t = −5.74, p = 0.00). Seven of 23 (30.4%) subjects in the WG were in a depressive state while only 1/15 (6.7%) patients in the RG had depressive status (HRDS-17 score 23). For the HAMA total score, the statistical result was also significant (9.3 ± 4.3 in the WG and 2.7 ± 4.1 in the RG, t = −4.74, p = 0.00) (**Table 1**). A total of 16/23 (69.6%) patients showed anxiety in the WG, while only one of those patients showed anxiety in the RG, with a score of 16.

### LT4 Withdrawal-Related Different Changes in Relative Cerebral Glucose Metabolism: WG Compared With RG/HG, Respectively

**Figure 2:**
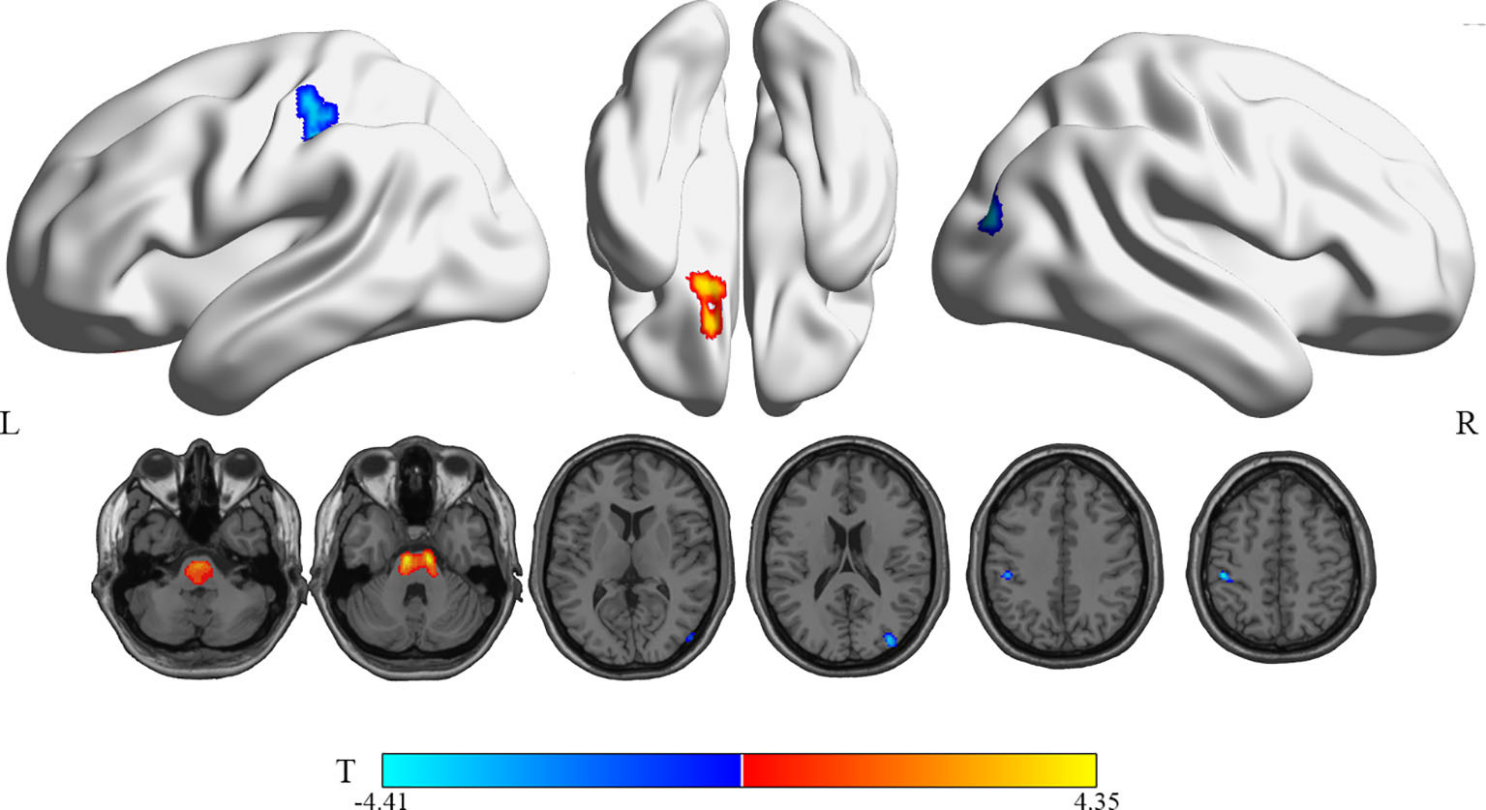
Brain regions with significant rCMRglu differences in WG compared with RG subjects. Compared with RG, glucose metabolism in WG patients decreased (cool) in the occipital_Mid_R and postcentral_L, but increased (warm) in the Frontal_Sup_Orb_L(uncorrected p-value <0.005; extend threshold = 150). The figure is depicted in neurologic orientation. The 3D render view and gray-scale image (a T1 structural MR image that is representative of MNI space). The color bar represents the T-value for each voxel.

Compared with the RG, patients in the WG showed significantly decreased ^18^F-FDG uptake in the right middle occipital gyrus (Occipital_Mid_R, MOG.R) and the left postcentral gyrus (Postcentral_L, PoCG.L) (uncorrected P < 0.005, cluster sizes >150; **Table 3** and **Figure 2**). The rCMRglu significantly increased in the left superior frontal gyrus, orbital part (Frontal_Sup_Orb_L, ORBsup.L).

**Table 3:** Brain Areas Showing Different rCMRglu between WG with RG or HG, separately.

Brain region	Side	Cluster size (voxels)	Coordinate (x, y, z)	Peak T-Value
**WG vs RG**				
Occipital_Mid	R	192	36 −84 20	−4.18
Postcentral	L	165	−48 −30 50	−4.41
Frontal_Sup_Orb	L	160	−14 14 −18	4.00
				
**WG vs HG**				
Postcentral	L	248	−48 −30 50	−3.98
Precuneus	L	255	−4 −64 64	−4.32
Temporal_Inf	L	271	−48 0 −46	3.96
Hippocampus	R	326	28 −32 −8	4.42

Results were uncollected at a p-value <0.005 at the voxel level, for clusters k >150 voxels^a^.

^a^WG, Levothyroxine withdrawal group; RG, Levothyroxine replacement group; HG, healthy control group.

The coordinates refer to the Montreal Neurological Institute (MNI) coordinate system; L, left; R, right.

**Figure 3:**
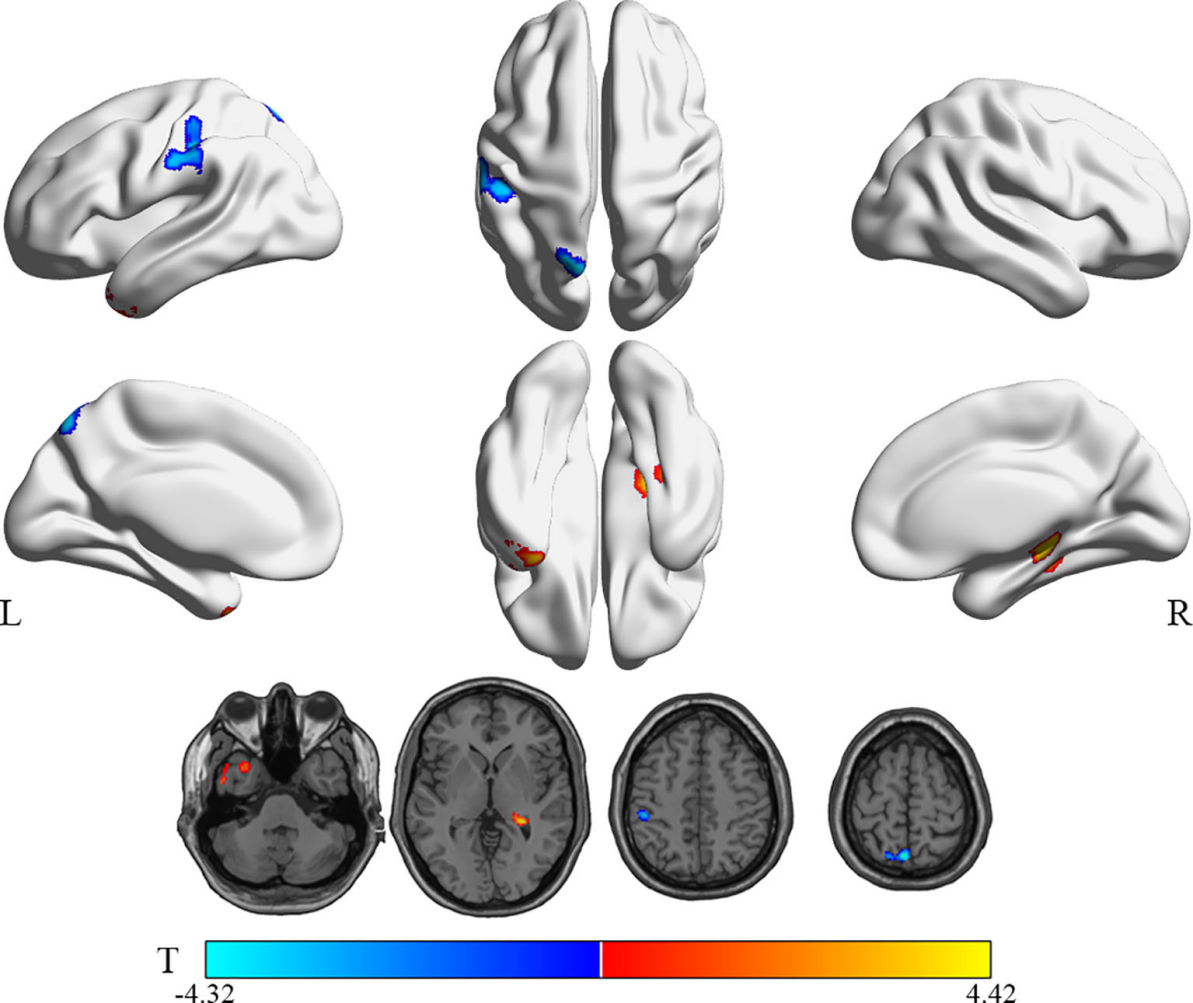
Brain regions with significant rCMRglu differences in WG compared with HG subjects. Compared with HG, glucose metabolism in WG patients decreased (cool) in the precuneus_L and postcentral_L, but increased (warm) in the Temporal_Inf_L and Hippocampus_R (uncorrected p-value <0.005; extend threshold = 150). The color bar represents the T-value for each voxel.

Compared with the HG, patients in the WG showed hyperactivity in the right hippocampus and the left inferior temporal gyrus (Temporal_Inf_L, ITG.L). In contrast, the rCMRglu demonstrated decreased metabolism in the left postcentral gyrus (Postcentral_L, PoCG.L) and left precuneus (Precuneus_L, PCUN.L) (uncorrected p < 0.005, cluster sizes >150; **Table 3** and **Figure 3**).

### Correlations of Thyroid Function (TSH, FT3, and FT4), Depressive (HRDS-17) and Anxious (HAMA) Symptoms With Relative Cerebral Glucose Metabolism in the WG

**Figure 4:**
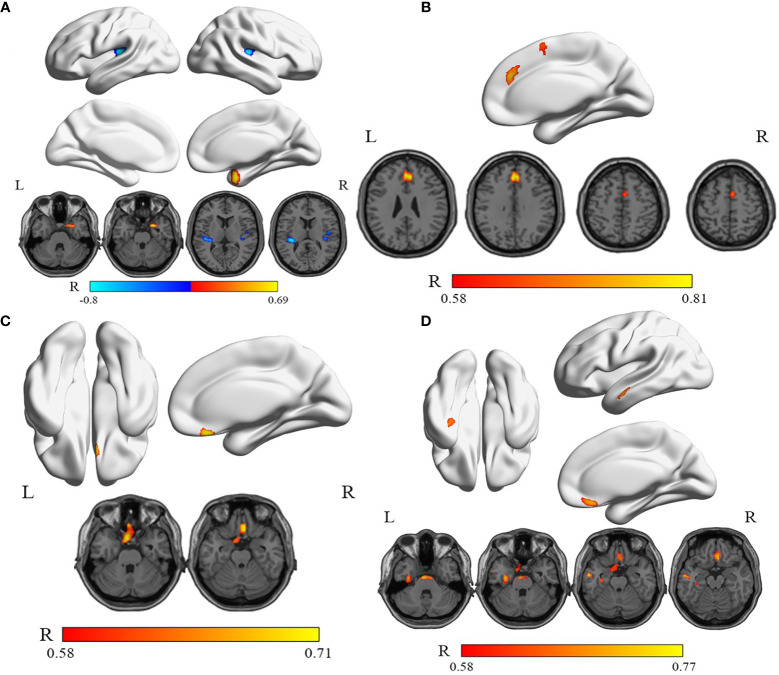
The correlations between serum TSH, FT4 levels, HRDS-17, HAMA scores, and relative rCMRglu in WG. **(A)** The rCMRglu in the left rolandic operculum and the right Heschel gyrus (cool) was a negative correlation with the serum TSH level, while the right parahippocampal gyrus (warm) was positively correlated. **(B)** The rCMRglu of the left medial superior frontal gyrus and the right supplementary motor area (warm) had a positive correlation with the serum FT4 level. **(C)** The glucose metabolism of the left gyrus rectus was positively correlated to the HRDS-17 score (warm). **(D)** The rCMRglu of the left fusiform gyrus, the left middle temporal gyrus, and the right gyrus rectus were positively correlated to the HAMA score (warm). The color bar represents the R-value for each voxel.

**Table 4** illustrates the correlations of thyroid function status, depression, and anxiety scores with relative rCMRglu in the WG. The correlation analysis shows a significantly positive correlation between the glucose metabolic activity in both the left superior frontal gyrus, medial (Frontal_sup_Medial_L, SFGmed.L) and the right supplementary motor area (Supp_Motor_Area_R, SMA.R) with the FT4 level (**Figure 4B**). But no significant correlations can be found between the free triiodothyronine (FT3) level with the glucose metabolic activity in any other gyrus. A negative correlation was found between the serum TSH level and the left rolandic operculum (Rolandic_Oper_L, ROL.L) and the right Heschel gyrus (HES.R), while the right parahippocampal gyrus (ParaHippocampal_R, PHG.R) was positively correlated to the serum TSH level (**Figure 4A**). The glucose metabolism of the left gyrus rectus (Rectus_L, REC.L) was positively correlated to HRDS-17 scores (**Figure 4C**). And the HAMA score was positively correlated to the metabolic activity of the left fusiform gyrus (Fusiform_L, FFG.L), the left middle temporal gyrus (Temproral_Mid_L, MTG.L), and the right gyrus rectus (Rectus_R, REC.R) (**Figure 4D**).

**Table 4:** The correlation of thyroid function (TSH, FT3, and FT4), depressive (HRDS-17) and anxious (HAMA) symptoms with relative cerebral glucose metabolism in the WG.

Brain region	Side	Cluster size (voxels)	Coordinate (x, y, z)	Peak R-Value
**TSH**				
ParaHippocampal	R	188	22, 6, −26	0.69
Rolandic_Oper	L	372	−36, −28, 16	−0.80
Heschel	R	156	38, −26, 14	−0.68
				
**FT3**				
N/A	N/A	N/A	N/A	N/A
				
**FT4**				
Frontal_Sup_Media	L	483	−2 38 32	0.81
Supp_Motor_Area	R	215	4, 5, 60	0.67
				
**HRDS-17**				
Rectus	L	566	−2, 34, −26	0.71
				
**HAMA**				
Fusiform	L	318	−32, −14, −28	0.71
Temproral_Mid	L	158	−56, −8, −20	0.74
Rectus	R	399	4, 30, −18	0.72

Results were uncollected at a p-value <0.005 at the voxel level, for clusters k >150 voxels^a^.

^a^WG, Levothyroxine withdrawal group.

The coordinates refer to the Montreal Neurological Institute (MNI) coordinate system; L, left; R, right; HRDS-17, 17 items of Hamilton Depression Rating Scale; HAMA, Hamilton Anxiety Rating Scale; FT4, free thyroxine; TSH, thyroid-stimulating hormone; N/A, Not Applicable.

## Discussion

This study mainly observed that even 4 weeks of LT4 withdrawal could lead to mood disorders and several brain regions of abnormal glucose metabolisms in PTC patients in China. Firstly, we observed patients in the WG with higher HRDS-17 and HAMA total scores, meant even transient hypothyroidism caused depression and anxiety. Secondly, the brain glucose metabolism of WG was compared with RG and HG, respectively. The results indicated that patients in the WG exhibited significantly decreased rCMRglu in the right occipital and the left postcentral gyrus, while increased in the left frontal gyrus. When compared with HG, in addition to the left posterior central gyrus, the glucose metabolism of the left precuneus was decreased, and the metabolism of the left temporal gyrus and the right parahippocampal gyrus increased. Thirdly, we identified some special regions in the WG that were related to the thyroid function (TSH and FT4) and depressive (HRDS-17)/anxious (HAMA) symptoms. This study confirmed that even after 4 weeks of LT4 discontinuation, the patients were at risk of neuropsychiatric disturbances. And several brain regions were affected by the thyroid dysfunction. Therefore, the regions had possible correlations with the transient thyroid dysfunction and mood disorders in this study.

Firstly, this study indicated that even temporary hypothyroidism could cause abnormal mood status in PTC patients. In clinical, we observed patients in the WG with higher HRDS-17 and HAMA scores. The results consisted with most studies, which reported that thyroid dysfunction indeed caused depressive and anxiety symptoms (4, 17–21). Therefore, this study has reconfirmed that hypothyroidism, even for a short duration, can cause abnormal mood swings and lead to increased risks of severe disorders such as depression and anxiety. Based on our conclusion, we recommend that neuropsychiatric tests including cognitive functions be used in patient management practices especially when LT4 withdrawal is involved.

Further, the brain glucose metabolism of the patients in WG was compared with RG/HG, respectively (**Table 3** and **Figures 2** and **3**). It is worth pointing out that several brain regions in which PTC patients with transient thyroid dysfunction reliably exhibited hypo/hypermetabolic compared with RG and healthy controls.

1. The decreases of rCMRglu in the MOG.R/PoCG.L (WG *vs* RG) and the PCUN.L/PoCG.L (WG *vs* HG) found in this study are generally in line with the previous neuroimaging studies carried out on hypothyroid patients. Decreased patterns of brain metabolism or CBF parietal-occipital areas, including postcentral gyrus, have been discussed (4, 5, 7, 19, 22). When compared with HG, another finding was the precuneus, an important role in the default mode network (DMN). Raichle demonstrated the DMN had high levels of metabolic activity during wakefulness, including the cingulate, precuneus, and parietal regions (15). According to our findings, among PTC patients with hypothyroidism, even for a short time, the left precuneus (important seed in DMN) showed lower rCMRglu. Therefore, the right occipital gyrus, the postcentral gyrus, and the DMN (especially the precuneus) may be cores of the potential target of these transient hypothyroidism-related mood disorders in hypothyroid PTC patients.

2. The hypermetabolism found in this study related to the ORBsup.L (WG *vs* RG) and the right hippocampus/the ITG.L (WG *vs* HG) seem to be special regions in transient hypothyroidism. The ORBsup.L is activated by both pleasant and unpleasant words and is associated with the posterior cingulate cortex. That means the importance of ORBsup.L in normal emotional processing, participating in clearly emotional/motivational executive functions (23, 24). Besides, we found increased metabolism in the right hippocampus and the left temporal gyrus in comparison with healthy controls, both of these regions may be related to anxiety (25, 26). An fMRI study identified reversible hypoperfusion in the anterior and posterior cingulate cortex, amygdala, and hippocampus in previously untreated hypothyroidism (27). Several brain regions were affected by thyroid dysfunction. We recommend that further studies on the relations between transient thyroid dysfunction and mood disorders continue to target these key regions.

Besides, we identified correlations between brain activities in certain cerebral regions, the thyroid functions (TSH and FT4), and the depressive (HRDS-17)/anxious (HAMA) symptoms in the WG. Multiple brain regions exhibited either positive or negative relationships. As we all know, thyroid hormones can regulate gene transcription through nuclear receptors, but the level of thyroid hormones in the brain cannot be measured. For example, Homan indicated that some key regions are sensitive to the low levels of hormones (28).

This study has limitations. Firstly, it was a cross-sectional study and therefore had a relatively small sample size. There are biases in PTC patients and healthy controls selection (six healthy subjects suffered from thyroiditis but in euthyroid status). Secondly, the connections in the whole brain are complex, and rCMRglu measures only one aspect of brain metabolisms. For example, if fMRI data were added, it could shed more light on the brain’s response to transient thyroid dysfunctions. Thirdly, the dynamic relationship between the level of peripheral thyroid hormone and the level of the cerebrospinal fluid, which directly affects abnormalities in the brain, was not clear. Moreover, other factors such as the hypothalamus-pituitary-thyroid (HPT) axis can also cause mood disorders. For example, in another study by our team, it was common to find high physiological FDG uptake of the pituitary in DTC patients (29).

## Conclusion

This is the first clinical study of the rCMRglu in age, gender, education, BMI, pTNM stage, and risk category-matched hypothyroidism DTC patients caused by the withdrawal of LT4 for 4 weeks in mainland China. In such hypothyroid status, patients showed depression and anxiety symptoms. We suggested more attention should be paid to these hypothyroid patients while they were in the hospital. Furthermore, such short time hypothyroidism may induce abnormal rCMRglu in the brain of DTC patients. Our findings may play a role in a deeper understanding of these transient hypothyroidism-related mood disorders. Of course, the current findings need to be further validated in a longitudinal study.

## Data Availability Statement

The raw data supporting the conclusions of this article will be made available by the authors, without undue reservation.

## Ethics Statement

The studies involving human participants were reviewed and approved by the Ethics Committee of Xin Hua Hospital Affiliated to Shanghai Jiaotong University School of Medicine. The patients/participants provided their written informed consent to participate in this study.

## Author Contributions

S-qW, Y-fY, and HW designed the study. FF, H-lF, and R-jZ collected clinical data. J-wS and X-zJ conducted the statistical analysis. S-qW drafted and wrote the manuscript. X-zJ, Y-fY, and WH supervised and edited the manuscript. All authors contributed to the article and approved the submitted version.

## Funding

This study was funded by the National Natural Science Fund of China (grant numbers 81671717, 81974269, 81974270, 51703126, and 51673116), and “Biomedical-engineering Cross Fund” of Shanghai Jiao Tong University (grant number YG2019QNA39).

## Conflict of Interest

The authors declare that the research was conducted in the absence of any commercial or financial relationships that could be construed as a potential conflict of interest.
